# Allogeneic Mesenchymal Stromal Cells as a Global Pediatric Prospective Approach in the Treatment of Respiratory Failure Associated with Surfactant Protein C Dysfunction

**DOI:** 10.3390/children10010162

**Published:** 2023-01-14

**Authors:** Gloria Pelizzo, Maria Antonietta Avanzini, Stefania Croce, Anna Mandelli, Elisa Lenta, Andrea Farolfi, Chiara Valsecchi, Salvatore Zirpoli, Giulia Lanfranchi, Eleonora Durante, Elena Zoia, Gianvincenzo Zuccotti, Valeria Calcaterra

**Affiliations:** 1 Department of Biomedical and Clinical Science, University of Milan, 20157 Milan, Italy; 2 Pediatric Surgery Department, Buzzi Children’s Hospital, 20154 Milan, Italy; 3 Pediatric Hematology Oncology, Cell Factory, Fondazione IRCCS Policlinico S. Matteo, 27100 Pavia, Italy; 4 Intensive Care Unit, Buzzi Children’s Hospital, 20154 Milan, Italy; 5 Pediatric Department, Buzzi Children’s Hospital, 20154 Milan, Italy; 6 Pediatric Radiology Unit, Buzzi Children’s Hospital, 20154 Milan, Italy; 7 Department of Internal Medicine, University of Pavia, 27100 Pavia, Italy

**Keywords:** allogenic, mesenchymal stromal cells, respiratory failure, surfactant protein C dysfunction, children, infusions

## Abstract

Mesenchymal stromal cells (MSCs) have been proposed as a new therapeutic strategy to treat congenital and acquired respiratory system diseases. We describe a case report of an 18-month-old male patient with progressive chronic respiratory failure, associated with mutations of the surfactant protein C gene (SFTPC) due to c.289G > T variant p.Gly97Ser (rs927644577) and c.176A > G variant (p.His59Arg), submitted to repeated intravenous infusions of allogeneic bone marrow (BM) MSCs. The clinical condition of the patient was monitored. Immunologic studies before and during MSC treatment were performed. No adverse events related to the MSC infusions were recorded. Throughout the MSC treatment period, the patient showed a growth recovery. Starting the second infusion, the patient experienced an improvement in his respiratory condition, with progressive adaptation to mechanical ventilation. After the third infusion, five hours/die of spontaneous breathing was shown, and after infusion IV, spontaneous ventilation for 24/24 h was recorded. A gradual decrease of lymphocytes and cell subpopulations was observed. No variations in the in vitro T cell response to PHA were determined by MSC treatment as well as for the in vitro B cell response. A decrease in IFN-γ, TNF-α, and IL-10 levels was also detected. Even though we cannot exclude an improvement of pulmonary function due to the physiological maturation, the well-known action of MSCs in the repair of lung tissue, together with the sequence of events observed in our patient, may support the therapeutic role of MSCs in this clinical condition. However, further investigations are necessary to confirm the result and long-term follow-up will be mandatory to confirm the benefits on the pulmonary condition.

## 1. Introduction

Cell-based therapy represents a new approach in regenerative medicine [1,2]. In particular, mesenchymal stromal cells (MSCs) have gained great interest in congenital and acquired respiratory disease treatment [3]. MSCs show an innate self-renewal capacity, they maintain differentiation potential, into multiple tissue-forming cell lineages, even after in vitro expansion [4]. Additionally, MSCs can act on almost all immune cells, modulating inflammatory responses. These capacities make MSCs key players in pulmonary tissue injury repair [5]. It is reported that through the secretion of paracrine factors and bioactive macromolecules, MSCs may reduce fibrosis and may promote normal alveoli and pulmonary vessel development [6,7,8,9].

Lung surfactant is a complex mixture of proteins and phospholipids that lines the air–liquid surface of the lung alveolar epithelium, reducing surface tension to prevent alveolar collapse and enhance pulmonary compliance during breathing [10,11]. There are four major lung surfactant proteins: hydrophobic SP-B and SP-C function in the formation of the protein–lipid matrix that lines the alveolar epithelium, while hydrophilic SP-A and SP-D function in innate immunity. Pathogenic variants in the genes that encode SP-B and SP-C result in surfactant dysfunction disorders, causing interstitial lung disease (ILD).

The clinical presentation and prognosis of ILD in children (chILD) are variable, ranging from mild nonspecific symptoms with complete recovery to a severe clinical picture, leading to chronic respiratory failure [3].

The standard chILD treatment is based on oxygen supplementation, and/or ventilation, and respiratory physiotherapy. Empirical medical therapy with anti-inflammatory and immunomodulatory drugs, such as corticosteroids, hydroxychloroquine, and azithromycin, is also proposed, with varying efficacy degrees. Lung transplant is a therapeutic option for pediatric patients with end-stage chronic respiratory failure. Preclinical and clinical models support a benefit of cell therapy to prevent the chILD progression [3].

Following the successful MSC-based treatment of a life-threatening respiratory syndrome associated with the filamin A (FLNA) gene mutation in a child [12], we performed this therapeutic approach as a rescue therapy in a child with chronic respiratory failure associated with SP-C dysfunction. The benefits of respiratory symptoms following repeated MSC infusions and immunologic studies are evaluated to support the MSC efficacy in vivo.

## 2. Patient and Methods

### 2.1. Patient

An 18-month-old male patient was hospitalized in our Pediatric Surgery Department with progressive chronic respiratory failure related to mutations of the surfactant protein C gene (*SFTPC*) due to c.289G > T variant p.Gly97Ser (rs927644577) and c.176A > G variant (p.His59Arg).

The child was born full-term, with no perinatal complications (39 weeks gestation birth weight 3370 g, APGAR 10 at 1 and 5 minutes, with an uneventful first month of life).

At 40 days, he was hospitalized for respiratory distress in respiratory syncytial virus bronchiolitis, and he was discharged without complications.

Two months later, growth failure and repeated respiratory virus infections (Rhynovirus, Adenovirus) with respiratory distress were recorded. A worsening deterioration of general and respiratory conditions occurred, requiring non-invasive mechanical ventilation (continuous positive airways pressure, FiO_2_ max 0.4).

At the age of 6 months, the child showed a progressive chronic respiratory failure necessitating intubation and tracheostomy, with ventilation support one month later. Artificial nutrition with feeding tubes was started.

Marked interstitial prominence with alveolar disease was observed. Genetic testing was performed, showing the SP-C mutation with paternal segregation. No family history of lung disease was reported. Sanger sequencing excluded the presence of variant mutation in the brother, paternal grandfather, and paternal uncle.

At our first evaluation, at the age of 18 months, the clinical picture was characterized by general muscular hypotonia and growth failure (weight 8.6 kg, −1.8 z-score World Health Organization (WHO), length 75 cm, (−1.7 z-score WHO percentile). The tracheostomy tube provided a continuous ventilatory dependence for 24/24 h (APCV, IPAP 15 cm, H_2_O-EPAP 9 cm, H_2_O-FR 30 apm, I:E 1:1.5-Ti 0.80 esc-Vt 85 mL, O_2_ adjunctive 3–4 L/min) and an indwelling naso-gastric tube guaranteed a pump-assisted continuous enteral feeding. The patient was under multidrug palliative therapy (steroid, benzodiazepine, α_2_-adrenergic agonist, morphine, antibiotics) since the first months of life.

At admittance to our Pediatric Surgery Department, the child underwent clinical evaluation, including gastroscopy and bronchoscopy. A percutaneous endoscopic gastrostomy (PEG) tube positioning was performed to allow an intermittent feeding, to favor an adequate nutritional support, to restore the disrupted circadian rhythms, and to prevent the episodes of inhalation.

Due to severe chronic respiratory failure and a possible worse prognosis, we proposed to initiate rescue therapy with allogeneic bone marrow (BM) MSCs. After receiving detailed information on the experimental therapy, we obtained the parents’ written informed consent as well as authorization to publish the images.

### 2.2. Methods

#### 2.2.1. BM-MSC Expansion

MSCs were expanded from allogeneic BM under Good Manufacturing Practice protocols at the clean room “Cell Factory” (Italian medicine Agency authorization number aM-209/2017), Fondazione IRCCS Policlinico S. Matteo, Pavia.

BM was obtained from a healthy donor, undergoing BM donation for hematopoietic stem cell transplantation, after informed consent was obtained. Mononuclear cells, obtained from gradient centrifugation, were cultured in Dulbecco’s modified Eagle’s medium (D-MEM) (Euroclone, Life Technologies), with 5% virally inactivated platelet lysate (MultiPL; Macopharma). The medium was changed twice a week. MSCs at passage 3 were cryopreserved. MSCs were released according to the requirements for morphology, cell viability, proliferative capacity, phenotypic and genotypic identity, and sterility.

#### 2.2.2. Infusion

The local Institutional Review Board of our Hospital and the Agenzia Italiana del Farmaco (AIFA) (PROT 91007) approved the MSC administration.

After thawing, MSCs were resuspended in sodium chloride 5% human albumin. Five IV infusions (1 × 10^6^ MSCs/kg body weight) were administered, in particular the second was 2 months after the first, while the following was 6 months apart. The MSC dosage was based on doses administered in adults [13,14] and in other pediatric indications, such as hematological diseases [15]

Before each infusion, chlorphenamine maleate, 0.2 mg/kg, was administered.

#### 2.2.3. Immunological Parameters

##### Flow Cytometry Characterization

B and T cell subpopulations were analyzed by flow cytometry using monoclonal antibodies (all from Becton Dickinson, BD, Milan, Italy). In particular, we used anti-CD4, CD8, CD16, CD56, CD45RA, CD45RO, and CCR7 for T cell and anti-CD19, CD27, and IgD for B cell subpopulations, as reported in Table 1.

Cells were acquired with Facs-Canto II (BD) and analysis was performed by Diva Software (BD).

##### In Vitro T Cell Response to Mitogen

The lymphocyte T cell response was evaluated by tritiated thymidine (^3^H) incorporation of in vitro phytohemagglutinin-activated (PHA, Gibco Milan, Italy) peripheral blood mononuclear cells (PBMNC), as previously reported [13]. Briefly, 100,000 PBMNC were cultured in triplicate in RPMI (Gibco), 10% FCS (Euroclone), in the absence or presence of PHA. Eighteen hours after ^3^H addition at 0.5 uCi/mL, cells were harvested. Radioactivity was measured as count per minute (cpm) by the Micro-beta microplate counter (Perkin Elmer, Milan, Italy). Results were expressed as stimulation index (SI) = cpm stimulated/cpm unstimulated.

##### In Vitro Ig Production

The B lymphocyte response was evaluated by in vitro Ig production after cell culture with anti-CD40 and IL-10, as previously reported [16]. Briefly, 100,000 PBMNC were plated in the presence of 200,000 CDw32L transfected murine fibroblasts, 0.5 μg/mL of anti-human CD40L (Calbiochem-Sigma, Milan, Italy), and 294 ng/mL of IL-10 (R&D System, Milan, Italy). After 10 days of culture at 37 °C, 5% CO_2_, supernatants were collected and stored at −20 °C. IgG, IgA, and IgM were quantified by ELISA and the results were expressed as µg/mL/10^6^ B lymphocytes.

##### Plasma Cytokine Levels

Cytokine levels were quantified by ELISA (Duo set-pair antibodies, R&D Systems) following the manufacturer’s instructions. In particular, IFN-γ, TNF-α, IL-17, IL-1β, IL-6, and IL-10 were quantified. Results were expressed as pg/mL.

## 3. Results

### 3.1. Clinical Data

No acute adverse events related to the MSC infusions were recorded. Vital parameters and renal and hepatic function tests were normal after each infusion.

**Figure 1:**
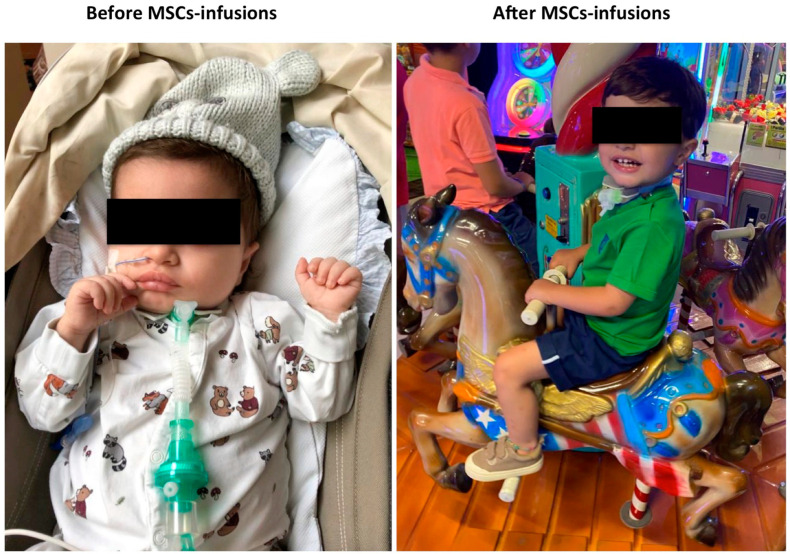
Clinical features before and after mesenchymal stromal cell infusions.

Throughout the MSC treatment period, the patient remained in good clinical condition, showing a growth recovery (BMI z-score −0.6 before infusion vs. +0.6 post-infusions, Table 2 and Figure 1). A significant improvement of neurological development was also noted (Table 2).

Before each infusion, the patient was submitted to a systematic review of stomas complications, including the possible removal of granulomas from the gastrostomy and tracheostomy, and surveillance of tracheal, esophago-gastric, and intestinal cultures, and no major accidents were recorded.

### 3.2. Respiratory Condition

In Table 3, the evolution of the patient’s respiratory parameters and therapies before and during the MSC treatments are shown. In addition, no systemic or respiratory infections were observed.

Starting the second infusion, the child experienced progressive improvement in his clinical respiratory condition. A progressive adaptation to mechanical ventilation, in the absence of respiratory exacerbation episodes, was adopted, maintaining an adequate exchange volume with a substantial reduction in inspiratory support. A reduction in trigger sensitivity was also obtained.

After infusion 3, five hours/die of spontaneous breathing was noted, and after infusion 4, spontaneous ventilation for 24/24 h was recorded.

Progressive steroid withdrawal was carried out, and after infusion 3, steroid therapy was stopped.

**Figure 2:**
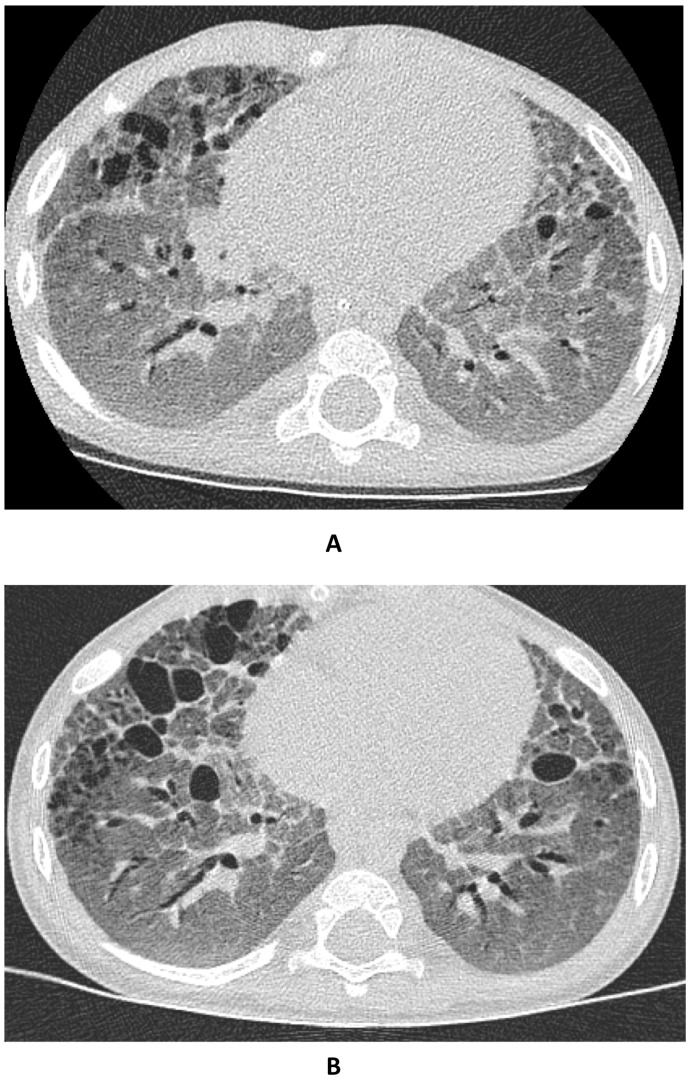
High-resolution CT imaging of pulmonary aspect before (**A**) and after (**B**) infusion 3 of mesenchymal stromal cells.

At imaging, before infusion, on chest X-ray, we observed changes of bilateral hyperinflation and diffuse granular parenchymal opacities marked in the basal regions. On the high-resolution CT (HRCT), we observed a pattern of diffuse ground-glass opacities (GGO) associated with interlobular septal thickening, reticular changes in a subpleural distribution, and multiple, variable-sized air-filled cystic lesions, especially in the anterior regions. The dilatation of the trachea and mainstem and segmental bronchi was also observed (Figure 2A).

On the last follow-up HRCT, performed before infusion 3, we observed a clear reduction of GGO, with an increase in the size and number of air-filled cystic lesions (Figure 2B).

**Figure 3:**
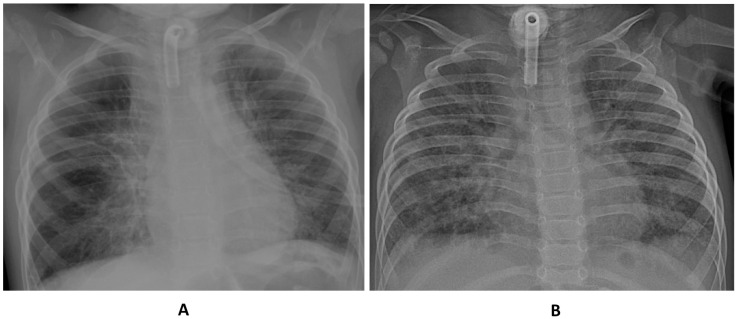
Chest X-ray pulmonary aspect before infusion 1 (**A**) and infusion 5 (**B**) of mesenchymal stromal cells.

Chest X-ray before infusion 5 showed a reduction of parenchymal opacities, mostly in the basal region (Figure 3).

### 3.3. Immunological Profile

Before each infusion, peripheral blood was collected, and plasma and PBMNC were separated and cryopreserved until analysis. The immunological profile was performed by evaluating T and B subpopulations. In particular, the number of naive (CD3+CD45RA+), memory (CD3+CD45RO+), and transitional (CD3+CD45RA+CD45RO+) T lymphocytes was defined, as well as naive (CD19+CD27^neg^IgD+), memory able to switch (CD19+CD27+IgD+), and memory switched (CD19+CD27+IgD^neg^) B cells. CD25+/FoxP3 T reg cells and CD16+ and CD56+ natural killer (NK) cells were also detected (Table 4).

Throughout the MSC treatment period, we observed a gradual decrease of the total number of lymphocytes, that for the recurrent infections and the use of immune-suppressive therapies, was higher than the upper limit for age. This trend was maintained for all the cell subpopulations. No variations in the in vitro T cell response to PHA were determined by MSC treatment, nor for the in vitro B cell response (Table 5). Regarding plasma cytokine quantification, we observed a decrease in IFN-γ, TNF-α, and IL-10 levels, while Il-17, IL-1β, and IL6 were not detectable at each time point (Table 6).

## 4. Discussion

Surfactant protein C (SP-C) dysfunction is a rare autosomal dominant disease due to a mutation in the SFTPC gene. It is associated with chronic respiratory failure and ChILD, with variations in the age of onset, severity, and clinical picture, ranging from respiratory distress immediately after birth to failure to thrive and progressive onset of respiratory failure at older ages [17,18,19].

Our patient showed a compound heterozygous variant of the SFTPC gene due to c.289G > T Variant p.Gly97Ser (rs927644577) and c.176A > G variant p.His59Arg (rs201567623). Severe clinical respiratory form has been described associated with homozygous mutation of c.289G > A in exon 3 of the SFTPC gene [20], confirming variable penetrance/functional effects. Even though the variants in SFTPC are not functionally investigated, considering the severe clinical course of the patient and the association between variants of the SFTPC gene and surfactant metabolism pulmonary dysfunction (OMIM#610913) in different severity forms, the role of this compound mutation in the respiratory failure has been considered and may be not excluded.

To date, no specific therapy is available. Anti-inflammatory and immunomodulatory therapies, including corticosteroids and hydroxychloroquine, have been described, with limited results on the evolution of the disease. Recent advances also offer opportunities to use gene therapies for genetic disorders of surfactant dysfunction. However, lung transplant remains the only option for survival beyond the first years of life [21,22].

MSCs show a great potential to proliferate, undergo multi-directional differentiation, and exert immunoregulatory effects. Their potential therapeutic role for respiratory inflammatory diseases has been described in adults [23].

Literature data support that the lungs may represent a target organ for MSCs. As reported, after intravenous infusion, MSCs first go to the lungs [24,25,26]. In an animal model, it has been shown that around 80% of the MSCs were found in the lungs within a few minutes after injection [24,25,26,27,28]. More recently, Ferrini et al. [29] demonstrated that MSCs could persist in the lungs for up to 28 days, independently of the delivery route.

To date, in the pediatric age, the effects of MSC treatments on lung disease have been described in bronchopulmonary dysplasia (BPD) [3]. A successful treatment in an infant with an FLNA gene mutation was previously reported by our group [12]. Here, we described the first report on the successfully repeated administration of intravenously delivered allogeneic BM-MSCs in an infant with a mutation of SFTPC and chronic respiratory failure, supporting that cell therapy could also be considered as a prospective approach to treat congenital respiratory diseases in pediatric populations. The benefits of MSCs on the surfactant dysfunction in the alveolar type II cells could also be considered. Jian-Dong Li et al. [30], in an in vitro study, showed that hBM-MSCs can differentiate into lung-specific type II pneumocytes, expressing SP-C. In another study, the therapeutic effects of BM-MSCs have been evaluated both in vitro and in vivo [8]. In vitro co-culturing with injured lung tissue increased the migration potential of BM-MSCs and the expression of SP-C [8].

In our patient, an improvement of general and respiratory condition starting at the second MSC infusion was noted, suggesting that serial administration instead of a single injection is necessary [12]. The improved clinical conditions allowed a good recovery in growth and neurological development. Changes in immunological parameters were also recorded, supporting an immunosuppressive MSC effect. The anti-inflammatory effects of the treatment seem to be confirmed by the decrease in IFN-γ and TNF-α levels, suggesting the control of the cytokine secretion from pro-inflammatory macrophages by MSCs [31].

Radiological findings from an initial homogeneous ground-glass attenuation to increasing signs of fibrosis with honeycombing, peri-bronchial, interlobular, and intralobular septal thickening, as well as cyst formation, were detected in our patient, and qualitatively similar observations have been made in other cohorts of affected patients [32,33,34].

Considering the worse, and potentially fatal, clinical situation of the patient, we proposed MSC treatment as a rescue therapy.

According to our previous experience and to data reported in the literature [13,14,15], we used a dosage of 1 × 10^6^ MSCs/kg, administered intravenously, taking into account that the lung, our target organ, represents the first-pass filter for MSCs. We would also underline that MSC infusions promoted, in addition to respiratory parameter improvement, the reduction of infections and, subsequently, of immunosuppressive therapy.

In the absence of a control period without an additional therapeutic option, it is not possible to define the final prognosis without cell-based treatment, and an improvement of pulmonary function without MSC infusions could also not be excluded. We cannot exclude that the progressive decrement of narcotic analgesics and immunosuppressive therapy and the physiological maturation of the respiratory and immune systems could be related to the improvement of the clinical condition. However, the well-known action of MSCs in the repair of lung tissue, promoting a normal alveoli and pulmonary vessel [6,9,35], together with the sequence of events observed in our patient, may support the therapeutic role of MSCs in this clinical condition. Long-term monitoring will be mandatory to define the beneficial effects of this treatment.

## 5. Conclusions

Our case report supports the role of MSC infusions as a prospective approach to treat respiratory failure associated with SP-C dysfunction in pediatric populations. The immunomodulant, antimicrobial, and reparative roles of MSCs should be proposed in the mitigation of lung injury; however, further investigations are necessary to confirm the results. Long-term follow-up will be mandatory to confirm the benefits on the pulmonary condition. Processes of pulmonary tissue regeneration, in patients affected with chronic pediatric lung diseases, pulmonary malformations, or pulmonary sequelae after surgery in the complex congenital malformations, such as congenital diaphragmatic hernia, remain not fully elucidated. The central paradigm in pediatric surgery remains the need to develop knowledge of all the mechanisms which underline regenerative pulmonary processes useful to support a pediatric surgical program. Allogeneic MSC treatment could be integrated in the pediatric surgical planning as a novel strategy to modify the poor prognosis.

## Figures and Tables

**Table 1:** Antigen surface expression considered for the definition of the T and B lymphocyte subpopulations.

Subpopulation	Antigen Surface Expression
T naive	CD3+CD45RA+
T memory	CD3+CD45RO+
T transitional	CD3+CD45RA+CD45RO+
	
CD4–CD8 naive T cells	CD4+CCR7+CD45RA+
	CD8+CCR7+CD45RA+
CD4–CD8 central memory T cells	CD4+CCR7+CD45RA-
	CD8+CCR7+CD45RA-
CD4–CD8 effector memory T cells	CD4+CCR7^neg^CD45RA-
	CD8+CCR7^neg^CD45RA-
CD4–CD8 Terminal effector T cells	CD4+CCR7^neg^CD45RA+
	CD8+CCR7^neg^CD45RA+
	
B naive	CD19+CD27^neg^IgD+
B memory able to switch	CD19+CD27+IgD+
B memory switched	CD19+CD27+IgD-

**Table 2:** Clinical parameters and nutritional support.

	Before Infusion 1	Before Infusion 2	Before Infusion 3	Before Infusion 4	Before Infusion 5
Age (years)	1 year and 9 months	1 year and 11 months	2 years and 5 months	2 years and 11 months	3 years and 4 months
Weight kg z-score	9.8 −1.7	10.3 −1.5	14 0.6	14 0	14.2 −0.2
Height cm z-score	80 −1.8	82 −1.7	87 −1.5	92 −1.3	94 −1.4
BMI kg/m^2^ z-score	15.3 −0.6	15.4 −0.5	16.4 1.5	16.6 0.8	16.1 0.6
Feeding	Exclusive enteral nutrition	Exclusive enteral nutrition	Exclusive enteral nutrition	Exclusive enteral nutrition	Enteral nutrition Introduction of liquid and creamy food
Neurodevelopmental assessments	General hypotonia Not autonomous sitting position No language	Improvement of general hypotonia Sitting position Rolling No language	Normal muscle tone Upright station with enlarged base and double support	Normal muscle tone Fine handling of objects Independent walking Initial phonation	Normal muscle tone Independent walking Phonation

**Table 3:** Patient’s respiratory parameters and therapies before each MSC infusion.

	Infusion 1	Infusion 2	Infusion 3	Infusion 4	Infusion 5
**Mechanical-Assisted ventilation**					
Time	24/24 h	24/24 h	19/24 h	0	0
Pressure	IPAP 16/EPAP 9/	IPAP 14/EPAP8/	IPAP 14/EPAP8/	-	-
FiO_2_	FR 22 0.5	FR 22 0.5	FR 22 0.45	1.5 L/min	1.5 L/min
Spontaneous ventilation	0	0	0	5 h/die	24 h
**Drugs**					
Prednisone	7.5 mg	6 mg	7.5 mg	stopped	stopped
Metilprednisolone	-	-	7 mg		
Lorazepam	0.5 mg	0.5 mg			
Clonidine	60 mg × 2	60 mg × 2	60 mg × 2	60 mg × 2	60 mg
Morphine	12.5 mg	12.5 mg	12.5 mg		
Melatonin	-	-	-	-	1 mg

**Table 4:** Cell subsets expressed as number of cells × 10^6^/mL. In vitro T cell proliferation to PHA is expressed as stimulation index (SI) = cpm stimulated/cpm unstimulated.

Number (×10^6^/mL)	Before Infusion 1	Before Infusion 2	Before Infusion 3	Before Infusion 4	Before Infusion 5
Total lymphocyte range (1.13–3.37)	7.89	7.44	5.88	4.59	3.46
CD3+	5.13	4.61	4.00	2.89	2.04
CD4+	3.55	3.05	2.12	1.79	2.01
CD25+/FoxP3	0.14	0.06	0.06	0.05	0.04
CD8+	1.50	1.41	1.47	0.87	0.48
CD19+	1.89	3.12	1.23	1.15	0.62
CD16+	0.08	0.07	0.06	0.05	0.03
CD56+	0.08	0.07	0.12	0.41	0.03
					
B naive	6.39	5.95	4.94	3.67	2.77
B memory able to switch	0.95	0.82	0.53	0.55	0.17
B memory switched	0.47	0.60	0.29	0.05	0.42
					
T naive	6.71	6.40	5.06	4.04	2.66
T memory	0.95	0.89	0.65	0.41	0.52
T transitional	0.08	0.15	0.06	0.05	0.00
					
CD4 naive	5.60	5.21	4.76	3.76	2.56
CD4 central memory	1.42	1.49	0.82	0.60	0.66
CD4 effector memory	0.63	0.52	0.24	0.14	0.17
CD4 terminal effectors	0.24	0.22	0.06	0.09	0.10
					
CD8 naive	6.23	5.65	3.88	3.72	2.08
CD8 central memory	0.08	0.15	0.06	0.18	0.10
CD8 effector memory	0.24	1.34	0.41	0.14	0.35
CD8 terminal effectors	1.34	0.37	1.47	0.55	0.90
					
T cell proliferation	305	317	155	309	210

**Table 5:** B cell response evaluated by in vitro Ig production. Results are expressed as ug/mL/10^6^ B lymphocytes.

	IgG		IgA		IgM	
	Resting	Activated	Resting	Activated	Resting	Activated
Before infusion 1	0.25	169	1.35	38	16	219
Before infusion 2	0.12	39	0.2	7	0.6	41
Before infusion 3	0.2	62	0.5	30	0.2	145
Before infusion 4	0.16	102	2	38	1	164
Before infusion 5	3.5	200	0.5	95	2	177

**Table 6:** Plasma cytokine levels. Results are expressed as pg/mL.

	Pre-Infusion 1	Pre-Infusion 2	Pre-Infusion 4	Pre-Infusion 5
IFN-γ	498	228	226	185
TNF-α	25	6	6	<2.5
IL-17	<3	<3	<3	<3
IL-1β	<1	<1	<1	<1
IL-6	<0.3	<0.3	<0.3	<0.3
IL-10	81.5	58	40	5

## Data Availability

Not applicable.
